# Push button replication: Is impact evaluation evidence for international development verifiable?

**DOI:** 10.1371/journal.pone.0209416

**Published:** 2018-12-21

**Authors:** Benjamin D. K. Wood, Rui Müller, Annette N. Brown

**Affiliations:** 1 International Initiative for Impact Evaluation (3ie), Washington, District of Columbia, United States of America; 2 Department of Economics, University of Copenhagen, Copenhagen, Denmark; 3 Chief Science Office, FHI 360, Washington, District of Columbia, United States of America; King Saud University, SAUDI ARABIA

## Abstract

**Objective:**

Empirical research that cannot be reproduced using the original dataset and software code (replication files) creates a credibility challenge, as it means those published findings are not verifiable. This study reports the results of a research audit exercise, known as the push button replication project, that tested a sample of studies that use similar empirical methods but span a variety of academic fields.

**Methods:**

We developed and piloted a detailed protocol for conducting push button replication and determining the level of comparability of these replication findings to original findings. We drew a sample of articles from the ten journals that published the most impact evaluations from low- and middle-income countries from 2010 through 2012. This set includes health, economics, and development journals. We then selected all articles in these journals published in 2014 that meet the same inclusion criteria and implemented the protocol on the sample.

**Results:**

Of the 109 articles in our sample, only 27 are push button replicable, meaning the provided code run on the provided dataset produces comparable findings for the key results in the published article. The authors of 59 of the articles refused to provide replication files. Thirty of these 59 articles were published in journals that had replication file requirements in 2014, meaning these articles are non-compliant with their journal requirements. For the remaining 23 of the 109 articles, we confirmed that three had proprietary data, we received incomplete replication files for 15, and we found minor differences in the replication results for five.

**Conclusion:**

The findings presented here reveal that many economics, development, and public health researchers are a long way from adopting the norm of open research. Journals do not appear to be playing a strong role in ensuring the availability of replication files.

## Introduction

In May 2015, two of us, as part of the Replication Program of the International Initiative for Impact Evaluation, convened a group of critics, supporters and others with an interest in replication research for a one-day consultation event in Washington, DC on replication research for international development. To our surprise, one of the more lively discussions at the event centered on whether it is reasonable to expect that the vast majority of published empirical studies can be exactly reproduced. Simply put, is it fair to expect that original data and programming code from an article exist and can be used by a third party to easily reproduce the published results?

All present agreed that this kind of reproduction is the most basic replication question. Some argued that this expectation should be a given–that of course original authors always have the data and code to reproduce their work. Others expressed strong doubts about how frequently authors really can provide the required materials to reproduce the published findings. These doubters argued that replication research should focus, at least initially, on this very first line of verification. Empirical research on this kind of verification supports the views of the doubters. For example, McCullough, McGeary and Harrison found that only 14 of 69 articles with data in the *Journal of Money*, *Credit and Banking* archive could be replicated [1], and more recently Chang and Li could only replicate 22 of 67 articles for which they requested data and code [2]. In this article, we provide new empirical evidence on whether journal publications of experimental and quasi-experimental studies of interventions in low- and middle-income countries can be verified in this way.

### Definition

We call this concept of replication ‘push button replication’, as in, can you push the button and reproduce the published results [3]. McCullough calls it reproducibility [4]. However, the term reproducibility has also been used to mean other things, including what we call external replication, which is the test of whether you can implement the program or experiment on a different sample and reproduce the outcomes achieved with the first sample [5]. Clemens defines a reproduction test as “resampling the same population but otherwise using identical methods to the original study.” [6] This definition is also different from push button replication. Clemens’ definition of verification, “ensuring that the exact statistical analysis reported in the original paper gives materially the same results reported in the paper,” is also not the same as push button replication [6]. That definition, which focuses on testing the analysis as reported in the original paper, is what we and others call pure replication [7, 8].

Thus, in the interest of being as specific as possible, we use the term ‘push button replication’ or PBR for the test of whether the code archived or submitted by the original authors can be applied to the data archived or supplied by the original authors to produce the published results.

A necessary condition of push button replicability is data accessibility. Most studies that explore push button replication start with this question, that is, are the data and code used to produce the tables and figures in the published article, often called the replication files, available for replication purposes. In cases where journals host an archive or require that replication files be shared when requested, this is a question of compliance. For example, Chang and Li divide their sample into those published in journals requiring replication file accessibility and those published in journals that do not [2]. Even when not required by the journal, however, the accessibility of replication files is a necessary condition for an article to be considered replicable (or more precisely, third-party replicable) as argued by McCullough [4].

### Research question

In this paper, we explore whether experimental and quasi-experimental intervention studies, hereafter called impact evaluations, conducted in low- and middle-income countries are push button replicable. As Brown, Cameron and Wood argue, the value of replication should be the highest for research that is likely to have policy impact, even from a single study [7]. Impact evaluations of interventions in developing countries, where the need for evidence is high and the quantity and quality of evidence are still relatively low, can often be highly influential. We apply our research question to a sample of development impact evaluations drawn from ten journals that publish a large number of such studies. The studies in our sample are all similar in terms of testing or evaluating some kind of intervention in a low- or middle-income country and using identification strategies based on experimental or statistical counterfactuals. On the other hand, our sample has more diversity compared to other samples drawn for similar replication exercises, as the studies come from different academic disciplines. Most notably roughly half our sample come from public health journals.

### Literature review

Perhaps the best-known example of PBR is McCullough, McGeary and Harrison, hereafter MMH, who explored the replicability of papers in the *Journal of Money*, *Credit and Banking* [1]. They selected 192 papers published between 1996 and 2002 that use data and code to produce their results. MMH defined replication as the reproduction of the results of a paper and they used the data and code provided by the authors explaining, “We made minor alterations to data and code to try to get the code to run with the data, but we did not attempt major alterations.” This definition is consistent with how we implement PBR. They found that only 69 of 186 articles met the data archive requirement of the journal. Out of those 69, only 14 could be replicated using the information from the archive.

A more recent example is Chang and Li’s study, which looks at the replicability of 60 empirical articles in macroeconomics published from 2008 to 2013 [2]. Chang and Li selected articles both from journals that require data and code be made available for replication purposes and from journals that do not. For those journals that do require replication files, Chang and Li were able to obtain these files from the authors for 29 of the 35 papers, and for those that do not, they obtained replication files from only 11 of 26 papers (six had confidential data). For the papers with data and code, Chang and Li conducted replication studies following the definition of MMH. They labelled a replication as successful when they could qualitatively reproduce the key results of the paper, a criterion that they admit is loose. Overall, they were able to successfully replicate only 22 of the 67 articles using the provided replication files without contacting the authors for assistance.

Galiani, Gertler, and Romero, hereafter GGR, examined a sample of 205 studies from nine economics journals [9]. Their concern is whether the replication files are publicly posted and whether the replication files meet their four requirements: raw data used in the study, final estimation data set, data manipulation code used to create the final estimation set from the raw data, and estimation code used to produce the final tables and figures. For their sample, they found that only a third of the articles had the raw data posted, while three quarters had at least one of the four types of replication files posted. Giving themselves a time limit of four hours per study, they tested those with publicly posted files and found that only 14% of the articles in the sample of 203 were fully replicable (from raw data to final tables and figures) and only 37% were partially replicable (from estimation data to final tables and figures).

Alsheikh-Ali, et al. focus just on the question of data accessibility [10]. They drew a sample of 500 articles comprising the first ten original research articles published in 2009 in the journals with the top 50 impact factors. Because the impact factor is calculated with a preference to the publication processes for basic science and health, all of the journals selected are of those types. Of the 500 articles, 351 were subject to some kind of data policy. Of the 351, 208 did not fully comply with the data policy of the journal where they were published, and only 47 deposited full primary raw data online. Of note, not one of the 149 articles in journals without requirements had full primary data publicly available online.

Savage and Vickers look specifically at replication file sharing by authors publishing in PLOS journals [11]. They requested the replication files from 10 papers published in either *PLOS Medicine* or *PLOS Clinical Trials*. They only received one data set. Their results documented that the PLOS data sharing requirements were largely unenforced at that time. Naudet, et al. select all 37 randomized controlled trials published by *The BMJ* and *PLOS Medicine* in the time since each journal adopted data sharing policies [12]. Naudet, et al. audit each article for whether the data were made available upon request and whether they could reproduce the published results using the provided data and the methods section of the published article. They find that 17 articles met their definition of data availability and of those, 14 were reproducible for all primary outcomes.

Wicherts, Bakker and Molenaar approach the question from a behavioral perspective rather than an audit perspective [13]. For 49 articles published in two major psychology journals they explore the relationship between the willingness to share the research data and the strength of the evidence and reporting errors in each article. Twenty one of the 49 corresponding authors shared some data. Their findings suggest that “statistical results are particularly hard to verify when reanalysis is more likely to lead to contrasting conclusions”.

In this paper, our subject of study is published studies, and our question is whether their key results are third party verifiable, meaning a third party can obtain the data and the program code (replication files) and run that code on the data to produce the same results published in the article. Our focus is not on a particular discipline, but rather on a particular type of research–development impact evaluations–for which even a single study can be policy influential. Although we look at compliance rates for journals with data sharing policies, our concern is the verifiability of evidence that may be used for policy making and programming. As such, we do not limit our research question to studies in journals that have data sharing policies. We also wanted to give each study the greatest possibility of being verifiable, so our process includes requesting replication files from authors, in addition to seeking public files. This audit design follows the example of Chang and Li [2].

## Methodology

We embarked upon this project in 2015 shortly after the consultation event mentioned in the introduction. While most authors are careful to support the concept of replication in public, in our experience many attack those who conduct it or fund it, so we knew we needed to tread carefully. Our first task was to establish a protocol for push button replication that would make these replication exercises replicable themselves and therefore make it clear that push button replication is intended to be a neutral test. Our second task was to set up the push button replication project to be transparent, again to reaffirm the intention of neutrality.

### The protocol

We developed the protocol over several months by writing a draft protocol, commissioning other researchers to pilot the protocol, and then revising the protocol based extensive discussions around what was learned in the piloting process. Our initial draft benefited from our experiences working with researchers conducting replication studies in the 3ie grants program. The protocol (see supporting information) includes several key features. It lays out specific sequential steps for the PBR researcher to take; it includes detailed checklists of tasks for preparing to run the code, running the code, and making the comparison; it requires a pre-registration of the key results; it provides a clear template for the final report; and it includes specific reporting requirements for each classification category.

The ordered steps include the communications with the original authors, and we provide templates for each of these messages. If the data and code for an article are publicly available, the protocol still requires the PBR researcher to notify the original authors that their article is included in our study and to share the PBR protocol with them. For cases where the data and code are not publicly available, the protocol provides a timeline for requests and reminders. Table 1 presents the possible classifications for PBRs as defined in the protocol.

**Table 1:** PBR classifications.

Classification	Definition
Comparable	Identical results or very small changes (like rounding)
Minor differences	Small differences in coefficients and/or p-values
Major differences	Meaningful differences in reported outcomes (especially in the key results) or the code does not reproduce published results
No access	The original authors do not reply or decline to provide data or code
Proprietary data	Unable to provide data but provided code and DSL documentation
Incomplete	Unable to reproduce part of the publication due to missing code and/or data

While McCullough argues that, given data and code, the classification of a replication outcome should be binary–either the code reproduces the published results exactly or it does not–our experience prior to this project suggested it is not always that straightforward [4]. If the dataset provided does not correspond exactly with the final dataset, e.g. limited observations were cleaned later in the analysis, there can be small differences in the coefficients or p-values. Even McCullough acknowledges that different versions of the same software package can yield different estimates. We have also learned over time that arguments about small differences do not benefit the larger objective of verifying findings for the purpose of informing policy. For those reasons, our protocol includes “comparable”, “minor differences”, and “major differences” classifications, and we avoid the use of the terms “successful” or “failure”.

The protocol provides guidelines for assessing the degree of differences between the results from the PBR and the published results. Our goal was to make this comparison objective. For statistical significance, the guidelines state that a difference in a p-value of 0.1 or greater should be considered a major difference; a difference in a p-value between 0.1 and 0.05 should be considered minor; and p-value estimates within 0.05 of each other should be considered comparable. The values of the parameters, or coefficient estimates, matter as well. We considered a decision rule using percentage change, but recognized that the meaningfulness of the size of parameters can be very different across studies precluding us from developing a single rule. Thus the protocol instructs the PBR researcher to use judgment, but to carefully document her decisions using summary statistics, like mean values, when available.

When we piloted the protocol on studies with dozens if not hundreds of published statistical results, it became clear that focusing on key results would help to make the process of comparing results both feasible and meaningful. We spent time considering whether we could use a quantitative decision rule such as a cutoff for the share of reported results that have minor or major differences, but that decision rule was complicated, if not impossible, to apply in practice. Chang and Li also focus on “key qualitative results” in making their determinations of successful replications [2]. We decided that the most credible way to focus on key results in determining a PBR classification is to publicly pre-register the key results for a study before a PBR on that study is initiated, and we incorporated this step into the protocol.

The protocol allows for the classification of incomplete to be matrixed. That is, the data and code can be incomplete but produce comparable results for the tables they do cover, or the data and code can be incomplete and produce differences in the results for the tables they do cover. If the original authors provided data and/or statistical code but we were unable to reproduce any of the findings from their paper, we coded the study as no access. If the data and statistical code provided allowed us to reproduce the results partially, even if only for some of the tables in the publication, we gave the study two codes, one for having incomplete files and one for the match between the PBR results and the results in the published article. In our analysis here, however, we use incomplete as the primary classification for an article; that is, the finding on data accessibility trumps the finding on comparability.

Once we finalized the protocol and embarked on the PBR project, the main features of the protocol stayed the same. However we made two changes during the course of the project that merit mention. These concern the comparison of statistical significance estimates and the requirement for writing and posting the key results. The change in statistical significance decision rules was driven by the difficulties we encountered when trying to recover p-values from code not originally designed to report them. Because of this problem, we allow for categorizing “one-star” level changes as minor differences and “two-star” level changes as major changes in statistical significance. For the key results, originally we required PBR researchers to read the article and write a key results document before contacting the original authors to request data. After spending the time to write up these documents numerous times and then not receiving data, we changed the protocol to require the write up of key results after the PBR researcher knows the data are available but before replication exercises begin.

### Transparency

The second task in developing the methodology for the PBR project was to make the processes transparent. We did this by setting up the project on the Open Science Framework (OSF). We began by posting all the relevant project documents, including the protocol, in a public folder on our OSF project page. We then set up separate spaces for each of the articles in the sample. Once a PBR researcher obtained the data and code (or was confident she would) for an article, she wrote the key results document and posted that in a public folder. In that way, the original authors, and anyone else, could see from the beginning what would be considered key results for the purpose of determining the PBR classification. Once the PBR was complete, the final report, which includes the classification, was posted in a folder accessible only to the project team and the original authors, where the key results documents now reside as well.

We publicly announced the launch of the PBR project in July of 2016 [3] and posted the PBR protocol on the 3ie website at that time.

### Process

After drawing the sample of studies, we divided them up among a group of PBR researchers including the three authors (with Wood and Müller each conducting a large number), some staff members at 3ie and several interns. One author, Müller, selected all the articles from the social science journals as a sub-sample with which to write his master’s thesis [14]. Each PBR was conducted by a single researcher, with a second researcher conducting a PBR for each study found by the first researcher to have major differences. We coded metadata from each study, such as sector and country, into an Excel spreadsheet along with the classification results. Figures were created using Excel, Stata, and R.

The expectation when one requests replication files is that original authors send ready-to-run code. If the code does not immediately run, the protocol requires researchers to “attempt to troubleshoot minor complications” in order to get the code to run but not to write new code. In practice, typical troubleshooting included running the code in an older version of the statistical software, ensuring all relevant user written packages were installed on the computer, and removing variables referenced in the code that were unavailable in the dataset. In a few instances we went beyond minor troubleshooting to change “use” commands to “merge” commands in Stata to allow the code to run, updating commands to the current version of the software, and even correcting typos in an attempt to reproduce the original results. The protocol does not limit the time researchers are allowed to spend on troubleshooting. When troubleshooting is not successful, the protocol requires PBR researchers to contact the original authors for assistance before making a PBR classification.

In all cases where we received data but the code would not run even after troubleshooting, or the data and code were not sufficient to reproduce all the published tables, or the data and code yielded major differences in the results, the PBR researcher contacted the original authors to give them the opportunity to provide an alternative dataset or code to reproduce the published results. If the code did not run on the data sent by the original authors, and they were not able to send code that runs on the data after follow up, we classified the PBR as no access. If the data and code only reproduced some of the tables, even after follow up, we classified the PBR as incomplete. The protocol specifies certain periods of time to wait for responses from the original authors, similar to the pre-specification of flowtime more recently advocated by Chang for replication research [15]. In practice these waiting periods were only minimums. PBR researchers often allowed for extra time or sent extra reminders.

In a few instances, we classified studies as having proprietary data. We created that classification to account for situations where the authors are unable to provide the data because they do not own them. To determine whether an article qualifies for the proprietary data classification, we required the authors to provide the estimation code and the contact information for the data owners. In each of these situations, we then contacted the owners of the data to request access for the purpose of the PBR, which also allowed us to confirm the authors’ claims that they could not provide the data.

In addition to coding a PBR classification for each study, we coded studies as having publicly available replication files or not at the time that the PBR for that study was initiated. We followed a number of steps to identify which studies had publicly available replication files. We first looked for data sharing statements in the articles, and determined whether they pointed to public replication files. We then searched both Harvard’s Dataverse and the corresponding authors’ personal webpages for public statements describing a process for accessing the replication files. In some cases the stated process required us to submit data confidentiality agreements, data use plans, or other documentation to gain access to these files. As long as a stated process exists for accessing the replication file, we coded the study as having a publicly available replication file. We coded all others as not having publicly available replication files, including those for which the data are proprietary.

We also coded the research funders acknowledged in each of the publications. We reviewed the funding statements and any other author notes included in the articles to identify the research funders. Many of the studies listed multiple funders, each of which we coded.

### Ethics statement

Ethical approval is not required. This investigation audits the availability of data and the computational accuracy of program code for published articles. All data provided to us were anonymized, and we only analyzed them by running the provided program code. See Naudet, et al. for a similar study not requiring ethical approval [12].

## The sample

This project was initiated under 3ie’s replication program, so our inclusion criteria for the sample reflect 3ie’s mandate. 3ie’s mandate is to increase the quantity and to improve the quality of impact evaluations of interventions implemented in low- and middle-income countries, what we call development impact evaluations for short. Our inclusion criterion for an impact evaluations requires a study to measure the net effect of an intervention using a counterfactual method. We include experimental designs (randomized controlled trials), natural experiments (or as-if random designs), fixed effects models, and observational studies using matching techniques. By development interventions, we mean experiments, projects or programs designed to improve human lives in low- and middle-income countries. While 3ie’s mandate focuses more on program effectiveness than on medical efficacy, our inclusion criteria capture a large number of public health studies of medical treatments tested in low- and middle-income countries.

We drew our sample from the top ten journals for development impact evaluations as determined using 3ie’s impact evaluation repository (IER). The IER is a database of metadata on development impact evaluations that have been identified using a systematic search and screening process designed to capture all such published studies. See Cameron, Mishra and Brown for a description [16]. We identified the top ten journals by looking for those that published the greatest number of development impact evaluations during the period 2010 through 2012 as catalogued the IER, which was complete through the end of 2012 at the time we started the project. Table 2 presents the top ten journals.

**Table 2:** The top ten journals for development impact evaluations.

Journal	Number development impact evaluations in IER 2010–2012	Number development impact evaluations in 2014	Public replication file requirement in 2014?	Replication file requirement in 2014?
**AIDS and Behavior**	17	6	No	No
**American Economic Journal: Applied Economics (AEJ: AE)**	22	8	No	Yes
**The BMJ**	13	8	No	No^
**Economic Development and Cultural Change (EDCC)**	15	4	No	No
**Journal of Development Economics (JDE)**	22	20	Yes	Yes
**Journal of Development Effectiveness (JDEff)**	21	4	No	No
**PLOS ONE**	21	34	No	Yes
**The Lancet**	25	3	No	No*
**Tropical Medicine and International Health (TMIH)**	14	3	No	No
**World Development (WD)**	25	19	No	No*

^Only required for RCTs of drugs or devices.

*Encouraged but not required.

As shown in Table 2, the top ten journals for development impact evaluations are quite diverse. Half are public health journals. Three are economics journals, and the other two are multidisciplinary development journals. Thus, this sample allows us to test push button replicability for similar types of research but across different academic disciplines. The total number of development impact evaluations published in these ten journals in 2014 is 109. In 2014 none of the public health journals had an open data requirement, and only one had a replication data requirement, the latter meaning that the journal requires authors to provide data and code for replication purposes upon request. Two of three of the economics journals had replication data requirements, and the two development journals did not.

## Results

### PBR classifications

**Fig 1:**
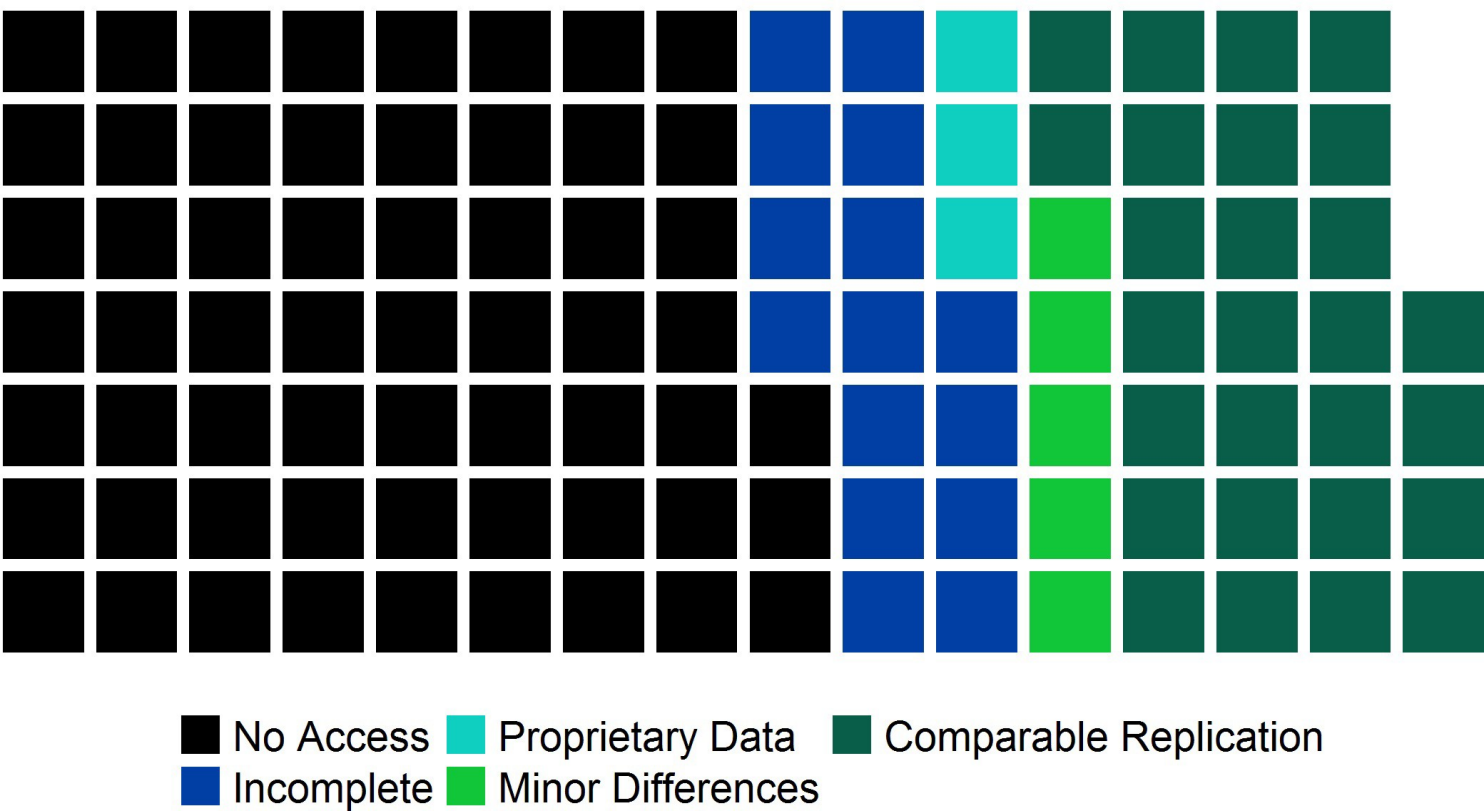
PBR classification results for entire sample. Each square represents one article, and the order of shading from left to right is no access, incomplete, proprietary data, (major differences), minor differences, and comparable.

Over half of the sample (59 out of 109) is classified as no access, 15 are incomplete, and another three articles have proprietary data (Fig 1). Thus, verification through full push button replication is not even possible for 71 percent of the articles in the sample. For those with complete data, there is none with major differences, five with minor differences, and 27 with comparable results.

**Fig 2:**
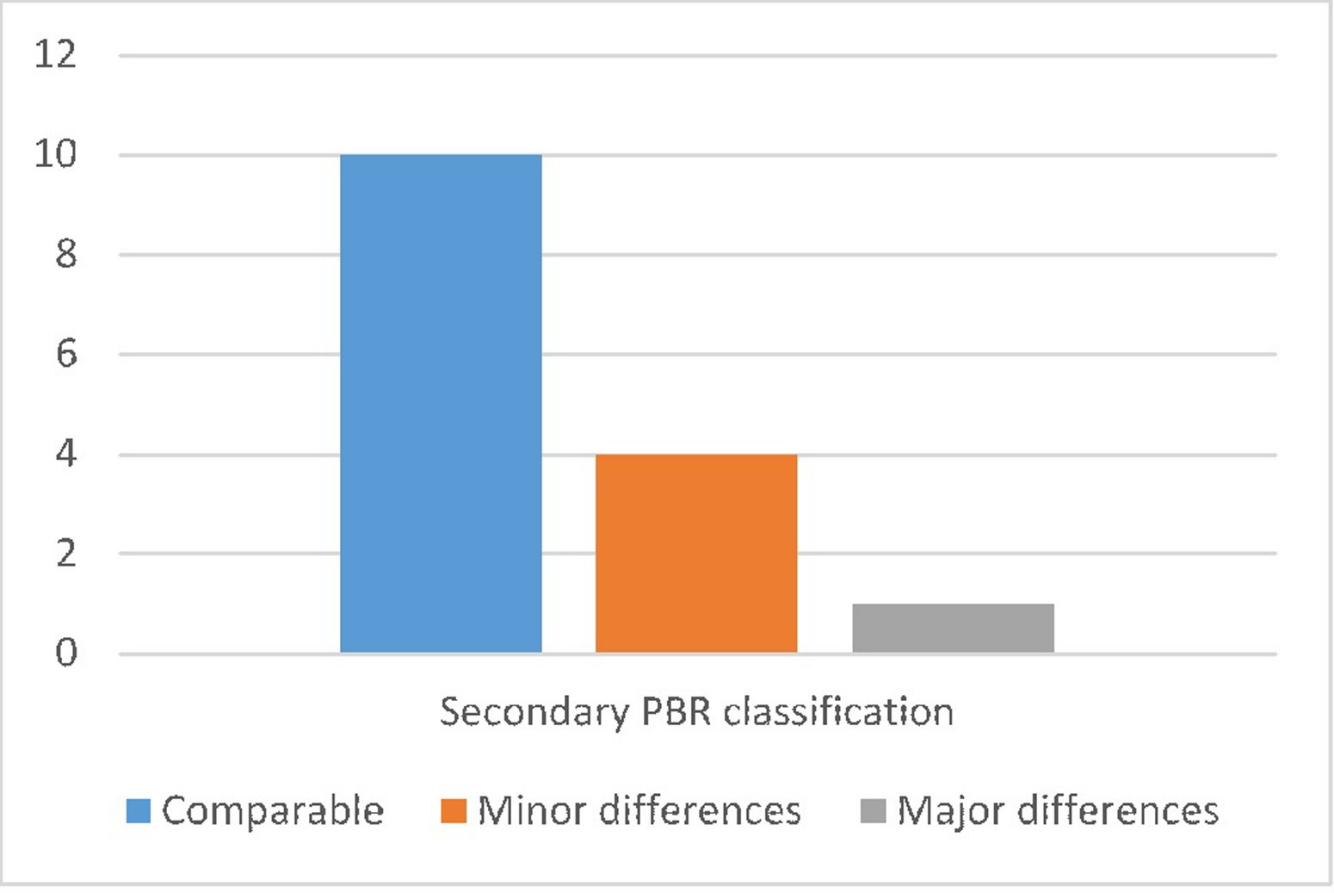
Secondary PBR classification of incomplete studies.

The replication files for the 15 incomplete studies in our sample produce a range of results. Two thirds of these studies have comparable results for the tables and figures we could produce (Fig 2). Four have minor differences, and one has major differences.

Table 3 presents the classification results by journal. The rows shaded in the table are for the journals that had replication data requirements in 2014.

**Table 3:** PBR results for the sample by journal.

Journal	Total number 2014	No access	Proprietary	Major differences	Minor differences	Comparable	Incomplete*
**AIDS and Behavior**	6	5	0	0	0	1	1
**American Economic Journal: Applied Economics**	8	0	2	0	0	6	0
**The BMJ**	8	3	0	0	1	4	2
**Economic Development and Cultural Change**	4	0	0	0	2	2	2
**Journal of Development Economics**	20	6	1	0	1	12	4
**Journal of Development Effectiveness**	4	3	0	0	1	0	0
**PLOS ONE**	34	24	0	0	2	8	2
**The Lancet**	3	3	0	0	0	0	0
**Tropical Medicine and International Health**	3	3	0	0	0	0	0
**World Development**	19	12	0	1	2	4	4
**Total**	109	59	3	1	9	37	15

* We also classify incomplete studies as comparable, minor, or major differences in the table

### Data access

Six teams of authors made a proprietary/restricted data claim that we rejected. Of those, five teams claimed they were unable to share the replication files with us because of institutional review board (IRB) or other data sharing limitations, even after we agreed to sign any data sharing confidentiality agreement they required. And one research team claimed a company controlled their replication file but were unable to document those restrictions for us or provide us contact information for requesting data access. For the three articles that do have proprietary data, we requested the data from the owners so that we could attempt a PBR using the code provided by the authors. We only received data from one, and only partial data at that. In the figures below, we exclude the three articles coded as having proprietary data from our analysis.

**Fig 3:**
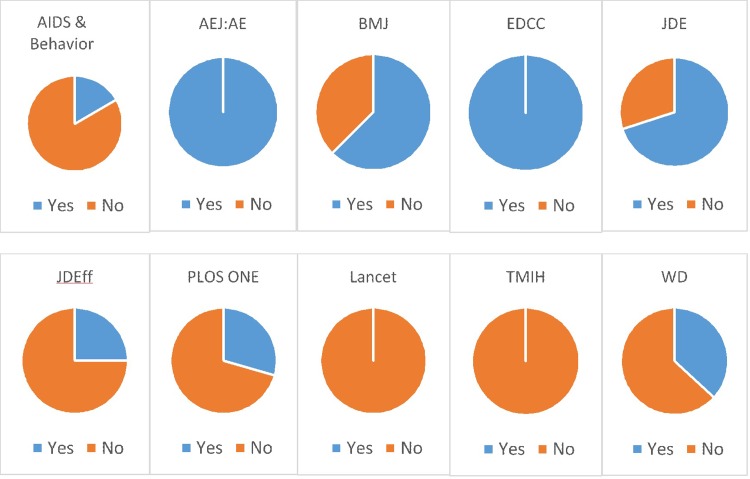
Accessibility of replication files through public access or by request, shares by journal. Those studies coded as ‘no’ include those with PBR classifications of no access and incomplete. Journal name abbreviations provided in Table 2.

Open data and code are rare in our sample. Only 14 of the 109 studies in our sample have publicly available replication files, and only four of the 20 articles published in JDE, the journal with a public replication file requirement in 2014, are compliant. For only four of the ten journals in our sample did we receive complete data and code for a majority of the sample articles (Fig 3).

Sixty-two of the 109 articles in our sample–those published in *JDE*, *PLOS ONE*, and *AEJ*: *AE*–are subject to a replication files requirement, either public or upon request. Only 26 articles of those 62 meet the requirement. Ten of the 20 JDE articles have no access or incomplete files. The authors refused to provide replication files for 24 of the 34 PLOS ONE articles in our sample, and two more provided incomplete files. The eight articles in AEJ:AE are fully compliant with the journal’s requirements, although two of those have proprietary data.

Ironically, many of the articles in our sample include statements to the effect that the authors will make the data available upon request, yet the authors refused to so this. We saw this frequently with *PLOS ONE* articles, revealing that the authors indeed understood the journal’s requirement when they published, however, seemingly had no intention of honoring it. We also occasionally saw such statements about making data available upon request in articles published in journals without such a requirement, but some of these authors also refused our request. One article’s authors stated in the publication that the data underlying their results are fully available without restriction but then responded to us that their data sharing and use agreement prevents them from sharing the data. On the bright side, our request alerted one group of authors that a replication file they thought was publicly available had actually not been uploaded. They promptly publicly released the replication file and thanked us for our efforts.

**Fig 4:**
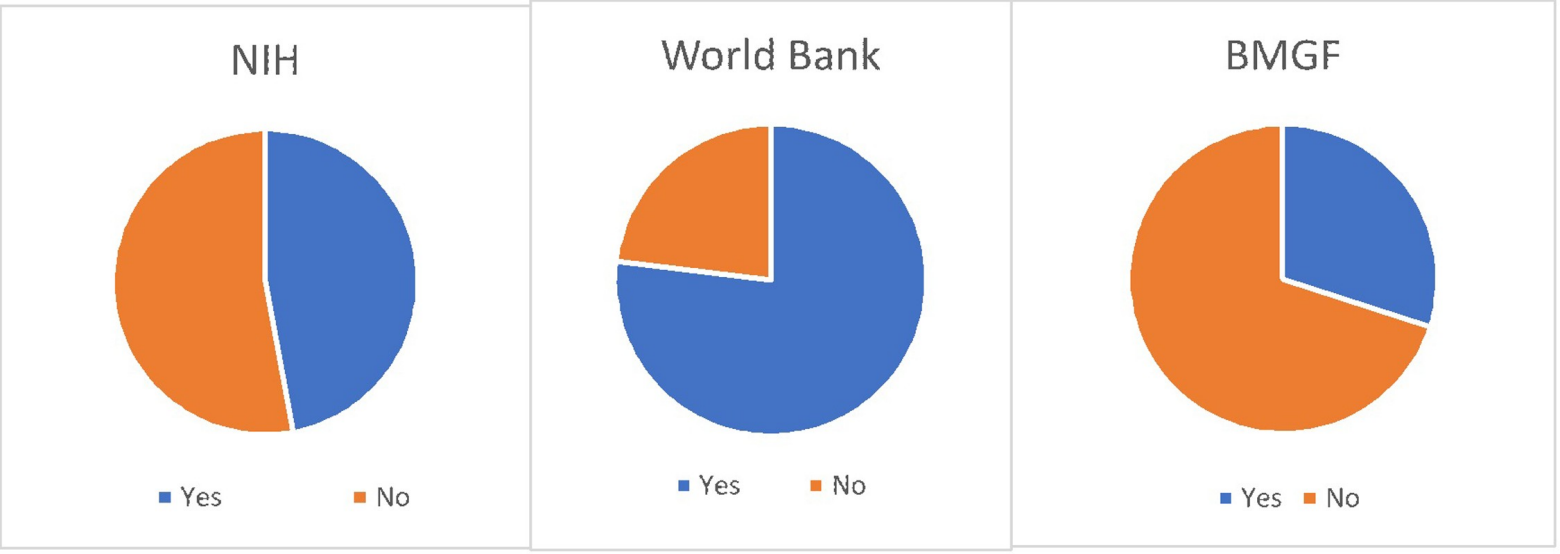
Provision of replication files, shares by three most prevalent research funders in the sample. Those studies coded as ‘no’ include those with PBR classifications of no access and incomplete.

Research funders also sometimes have open data or replication files requirements. These requirements can differ based on department or bureau within the agency, or according to specific grant or contract agreements, so we are unable to classify funders by specific policies. Nonetheless, it may be interesting for readers to see the data access results by funder. Ninety-five of the 109 studies in our sample report funding sources, including one study that reports “internal funds.” For these 95 articles, we coded 129 funders by name. The funders include universities, foundations, and public donors. Three funders appear in the sample ten or more times, with the next most prevalent funder appearing only five times. The U.S. National Institutes of Health (NIH) and its branches is the most frequently named funder in our sample with 17 articles. The World Bank has 13; Bill and Melinda Gates Foundation (BMGF) has 10. We received data and code for a majority of the World Bank’s sample articles, roughly half of NIH’s sample articles, and less than half of BMGF’s sample articles (Fig 4).

## Discussion

In our experience with push button and other kinds of replication research, we often hear authors complain that the cost of preparing and providing replication files is too high. Nevertheless, for several studies in our sample we found neatly written and commented code that correctly reproduced every table stored in easily accessible online databases. The economist David McKenzie co-authored five papers in our sample and all of them have data and code accessible online, and all received the classification “comparable replication” [17–21]. Olken, Onishi, and Wong was computationally very complex but the results could be reproduced by essentially pushing one button [22]. *AEJ*:*AE* had data and code available on their webpage for six out of six papers with non-proprietary data and all received a comparable classification.

Some observers argue that replication research is unnecessary given the movement towards open research. The idea is that open data or replication files requirements will motivate researchers to conduct more careful research from the beginning, so it will not be necessary to conduct replication studies to verify their published results. The findings presented here, however, reveal that many economics, development, and public health researchers are a long way from adopting the norm of open research. Not one of the papers in the five public health journals has publicly available replication files.

Authors provided a range of reasons for not providing replication files. A few examples include the inability to determine the final version of the statistical code, data use agreements that prevent any replication file sharing, and an unwillingness (or unresponsiveness) from the corresponding author to share the replication file. One researcher responded that the data and code were lost after a computer hard drive crash. Even when the data and code were provided, we encountered multiple instances when the code would not run. Unfortunately, when we contacted the authors about these problems they often did not reply. As we could not reproduce any of the results from these articles, we coded them as no access.

In the cases where the full replication files are available, the results are encouraging. The PBR classification for 84 percent of this subset is comparable and for the remaining 16 percent is minor differences. However, in addition to those articles for which no data are available, there are 25 articles in the sample with incomplete data, and among these there is one with major differences and four more with minor differences.

The results of our PBR project suggest that the biggest constraint to the push button replicability of published research, here represented by development impact evaluations, is the accessibility of replication files. We set the bar low, in that we made the effort to request the data and code, multiple times if necessary, rather than restricting our data question to one of open data as GGR do. We personally believe, however, that by 2016 or later, any data used to publish a study in 2014 should be publicly available except in extreme proprietary circumstances.

Funder requirement is one way of ensuring accessibility to replication files. Looking at the five most prevalent research funders in our sample, we find that replication files are available for the majority of the articles funded by the World Bank, USAID, and SIDA. On the other hand, the NIH did have in place requirements in 2014, including the NIH data sharing policy [23] and the NIH guidance for access to research data [24], and yet the majority of NIH-funded articles in our sample have a no access classification.

Journals do not appear to be playing a strong role in ensuring the availability of replication files. For example, there is no replication file access for over 70 percent of the articles published in *PLOS ONE*, which has one of the stricter data availability policies of the journals in our sample [25]. The *AEJ*:*AE* is the exception among our sample of ten journals. Six of six studies without proprietary data have publicly accessible files.

## Supporting information

S1 File (XLSX)
